# Designing supramolecular self-assembly nanomaterials as stimuli-responsive drug delivery platforms for cancer therapy

**DOI:** 10.1016/j.isci.2023.106279

**Published:** 2023-02-27

**Authors:** Yingqi Liu, Yunyun Wu, Zhong Luo, Menghuan Li

**Affiliations:** 1 School of Life Science, Chongqing University, Chongqing 400044, P. R. China; 2 Chongqing Municipal Center for Disease Control and Prevention, Chongqing 400042, China

**Keywords:** Biomaterials, Cancer, Drug delivery system, Molecular self-assembly, Supramolecular materials

## Abstract

Stimuli-responsive nanomaterials have attracted substantial interest in cancer therapy, as they hold promise to deliver anticancer agents to tumor sites in a precise and on-demand manner. Interestingly, supramolecular chemistry is a burgeoning discipline that entails the reversible bonding between components at the molecular and nanoscale levels, and the recent advances in this area offer the possibility to design nanotherapeutics with improved controllability and functionality for cancer therapy. Herein, we provide a comprehensive summary of typical non-covalent interaction modes, which primarily include hydrophobic interaction, hydrogel bonding, host-guest interaction, π-π stacking, and electrostatic interaction. Special emphasis is placed on the implications of these interaction modes to design novel stimuli-responsive drug delivery principles and concepts, aiming to enhance the spatial, temporal, and dosage precision of drug delivery to cancer cells. Finally, future perspectives are discussed to highlight current challenges and future opportunities in self-assembly-based stimuli-responsive drug delivery nanotechnologies for cancer therapy.

## Graphical abstract

**Figure:**
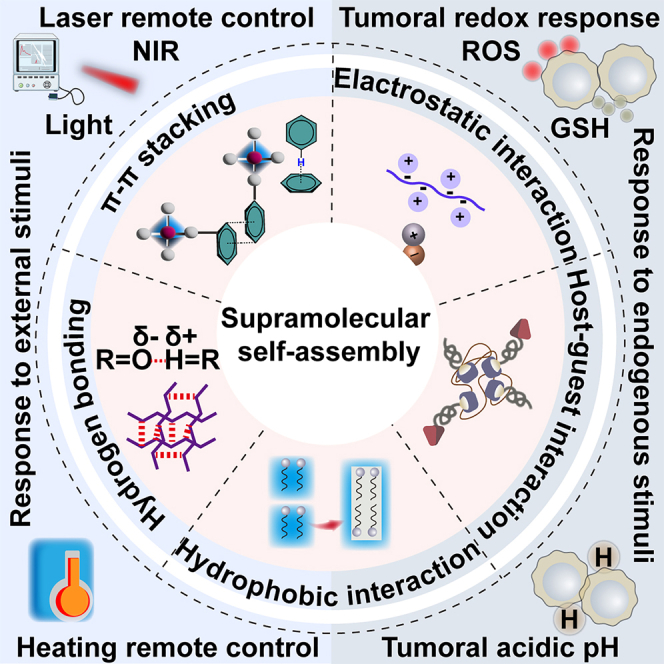

## Introduction

Chemotherapy is the mainstay of cancer treatment, which deploys cytotoxic agents to inhibit cancer cell growth for curative or palliative purposes.^1^^,^^2^ However, despite the continuous progress in the screening and development of novel anticancer agents, the performance of chemotherapy in clinics is still unsatisfactory due to multiple factors. Typically, systematically administered anticancer drugs are rapidly cleared from the body via metabolization, degradation, or excretion. Meanwhile, free drug molecules fail to adequately accumulate in desired sites of action, which not only compromises the antitumor potency but also induces severe off-target toxicities.^3^^,^^4^ Furthermore, it is impossible to regulate the pharmacokinetic properties of these molecular agents after entering the human body, which makes it difficult to maintain the tumor-specific drug concentration within the designated therapeutic window and significantly increases the risk of either overdose or insufficient response. Interestingly, stimuli-responsive drug delivery technology shows promise to overcome these challenges and improve the eventual therapeutic responses.^5^^,^^6^ Under ideal circumstances, these nanomaterials should effectively home to the tumor tissues and release their payload in response to certain exogenous or tumor-specific endogenous cues, thus improving the pharmacokinetic profiles *in vivo* to achieve balanced antitumor potency and safety.

The recent advances in supramolecular self-assembly technology provide emerging opportunities to develop advanced stimuli-responsive drug delivery nanosystems. Supramolecular self-assembly is a ubiquitous phenomenon in living organisms, which is defined as the spontaneous formation of complex architectures from basic molecular or nanoscale building blocks and is responsible for the maintenance of vital cellular structures as well as biochemical processes.^7^^,^^8^^,^^9^ In contrast to covalent synthesis, supramolecular self-assembly relies on those weaker non-covalent interaction forces including hydrophobic interaction, hydrogel bonding, host-guest interaction, π-π stacking, and electrostatic interaction to generate thermodynamically stable architectures with well-defined molecular patterns.^10^^,^^11^^,^^12^ Owing to the reversible nature of these interaction forces, supramolecular self-assembled nanostructures are in an intrinsically dynamic and flexible state, which are capable of rapidly responding to specific stimuli and changing their molecular patterns.^13^^,^^14^^,^^15^^,^^16^ Understanding the structural transformation processes of these supramolecular materials and the corresponding changes in their physical/chemical/biological properties is thus crucial for the development of novel anticancer nanotherapeutics, enabling on-demand tailoring of their pharmacokinetic properties for robust anticancer therapy.

Supramolecular nanotechnology has been extensively explored for biomedical applications and the accumulated progress in this area has been excellently reviewed in several previous studies. For instance, Liang et al. discussed the application of intratumoral cues such as reactive oxygen species (ROS), greater cytosolic glutathione (GSH), and acidity to trigger the *in situ* construction of supramolecular self-assemblies for theranostic purposes.^17^ Li et al. thoroughly discussed the molecular cooperativity between individual components in self-assembled nanosystems for improving diagnostic precision and therapeutic efficacy.^18^ Zhang et al. focused on the preparation of two-dimensional nanomaterials using organic components such as proteins, peptides, DNAs, etc, which could fulfill a broad spectrum of roles such as biosensors and biomedicines.^19^ However, while there are ample reports that exploit the unique stimuli-responsiveness of supramolecular self-assembly nanosystems for cancer therapy, there are few reviews that comprehensively analyze the therapeutic relevance of typical supramolecular self-assembly principles as well as their utility as guidelines for developing stimuli-responsive drug delivery systems. In this review, we aim to provide a concise yet comprehensive discussion on the significant advances in stimuli-responsive supramolecular drug delivery nanosystems. We summarize representative supramolecular interaction modes and thoroughly discuss their chemical characteristics in the context of cancer therapy. Extending from their unique chemical properties, we further discuss how they can be used to direct the construction of drug delivery nanosystems that could readily respond to various exogenous or endogenous stimuli. Furthermore, a perspective is added regarding the translational challenges encountered in previous studies and potential opportunities in this area.

## Molecular basis directing the formation of supramolecular self-assemblies

In this section, we discuss those representative non-covalent forces that govern the spontaneous arrangement and organization of molecular building blocks to form supramolecular self-assemblies, which primarily include hydrophobic interaction, hydrogel bonding, host-guest interaction, π-π stacking, and electrostatic interaction. Contrasting to the short distance (usually less than 0.2 nm) and high strength (above 50 kcal/mol) of covalent bonds, these non-covalent interactions are significantly weaker but could occur at much longer distances, underlying the molecular basis for the spontaneous formation and on-demand dissociation of long-range ordered architectures.^20^

### Hydrophobic interaction

Hydrophobic interaction is a common phenomenon in nature, which describes the fact that hydrophobic non-polar molecules are prone to aggregation in an aqueous environment.^21^^,^^22^ It is well established that the presence of non-polar molecules in water would perturbate the hydrogel bond network of surrounding water molecules and increase their free energy cost, thus the aggregation of the non-polar molecules becomes a thermodynamically favorable process as it minimizes the total free energy penalty.^23^ Hydrophobic effect is responsible for the formation of many cellular structures including lipid bilayers and chromosomes, as well as driving vital biological processes such as protein folding, ligand-receptor recognition, and liquid-liquid phase separation. Hydrophobic interaction has already been widely used to prepare nanobiomaterials for drug delivery applications. For instance, hydrophobic effects could drive the self-assembly of phospholipid molecules into water-stable lipid bilayer structures (liposomes), which have already entered clinical use for the treatment of multiple cancer indications.^24^^,^^25^^,^^26^^,^^27^ Alternatively, phospholipids could also be condensed into solid lipid nanoparticles with the assistance of additional surfactants, which have been used for the delivery of mRNA vaccines.^28^^,^^29^^,^^30^ Amphiphilic polymers with separated hydrophilic and hydrophobic blocks could self-assemble into micelles through the hydrophobic interaction-driven aggregation of the hydrophobic segments.^31^^,^^32^^,^^33^

Interestingly, hydrophobic effect-driven supramolecular self-assembly has demonstrated particular interest in the delivery of anticancer drugs as most of the existing anticancer agents are intrinsically hydrophobic. There are three general approaches for the efficient loading of hydrophobic anticancer drugs, which could be realized through (1) self-assembly of drug molecules into nanoaggregates, (2) insertion of drug molecules into hydrophobic domains, or (3) complexation of drug molecules onto hydrophobic surfaces. Nevertheless, it is notable that hydrophobic interaction is a non-specific effect and additional purification treatments are usually needed to prevent or remove undesirable products.

### Hydrogen bonding interaction

Hydrogen bonding is defined as the dipole-dipole attraction between a hydrogen atom (hydrogen-bond donor) covalently bonded to highly electronegative atoms, which are usually nitrogen, oxygen, or fluorine atoms, and another highly electron-rich atom (hydrogel-bond acceptor).^34^^,^^35^ The strength of hydrogen bonds is affected by the molecular geometry, solvent, and electronegativity of the acceptor atoms, which is usually in the range from 5 to 150 kJ/mol.^36^^,^^37^ A well-established example of hydrogen bonding-driven self-assembly in living organisms is the formation and stabilization of DNA double helix, for which two cDNA single-strands are held together by hydrogen bonding between the numerous nitrogenous base pairs.^38^^,^^39^ Unlike the hydrophobic effect described previously, hydrogen bonds are intrinsically directional and complementary, which is beneficial for generating nanostructures with well-defined geometry and architecture. Currently, hydrogen bonding-mediated self-assembly has already been employed to direct the complexation of polymeric units or ligand-modified nanoparticles. For instance, Zhou et al. developed a family of polyurea-based peptidomimetics with aggregation-induced-emission properties through the one-pot reaction of L-lysine ethyl ester diisocyanate, L-cystine dimethyl ester dihydrochloride, and L-lysine ethyl ester dihydrochloride, which could self-assemble into water-stable fluorescent nanostructures including nanovesicles and nanotubes through polyvalent interaction forces including hydrogen bonding, ionic interaction, and hydrophobic interaction.^40^ Moreover, by tuning the hydrogen bonding strength via altering trifluoroacetate levels in incubation media, the PU assemblies presented nanovesicle size expanding/shrinking or nanotube-to-vesicle deformation behaviors. Alternatively, Wei et al. exploited the selective hydrogen bonding between 4-hydroxythiophenol-functionalized Au nanoparticles and pyridine moieties in poly(styrene-b-2-vinyl pyridine) block copolymer templates to yield well-ordered Au-containing patterns on silica surface as a charge memory coating, and the memory window characteristics of the Au layer could be easily adjusted by changing the amount of Au nanoparticle precursors.^41^

### Host-guest interactions

Host-guest interaction describes the complexation of two structurally complementary components and is an important branch of supramolecular chemistry.^42^^,^^43^ From an overall perspective, the host-guest complex involves a larger molecular or nanoscale component (host) with a binding pocket and a smaller component (guest), which could be installed into the previously mentioned pocket and stabilized through non-covalent forces, eventually entering an interlocked state.^44^^,^^45^^,^^46^ The requirement of structure and binding complementarity for the successful inclusion of the guest component would lead to substrate specificity and selectivity to some degree, while the relatively weak strength of the attraction forces between the host and guest components allows the non-invasive dissociation of the interlocked complex in a reversible manner.^47^^,^^48^ The host-guest interaction is a common phenomenon in a biological environment. Indeed, almost all biomolecule recognition systems involve some kind of host-guest interaction mechanisms, which contribute to the molecular specificity of vital biochemical processes such as antigen binding, receptor recognition, and enzymatic reactions.

Based on the accumulated insights from previous reports, we have summarized two major strategies to develop anticancer drug delivery nanosystems through host-guest interaction mechanism. Notably, biocompatible macromolecules with natural docking pockets have already been widely used for the incorporation of hydrophobic small-molecule drugs, exemplified by the clinical success of various cyclodextrin-drug complexes.^49^^,^^50^ Drug molecules with appropriate size and shape could be interred into the hydrophobic central cavity of the cyclodextrin ring via hydrophobic interaction, and the resultant complexation would significantly improve their *in vivo* stability and availability. Moreover, the interlocked state could be disrupted in response to perturbations in ambient environment, leading to efficient drug release in a controllable manner. Instead of using drug molecules as guest molecules for self-assembly-enabled incorporation into macrocyclic rings, it is also possible to encapsulate drugs into porous nanoparticles and use rotaxane-based nanovalves to cap the pore openings.^51^^,^^52^ These rotaxane-based nanovalves are essentially macrocyclic rings treaded onto a molecular axis through host-guest interaction. Upon stimulation by specific triggering signals, the macrocyclic rings could shift along the molecular axis or completely detach from the nanoparticle surface, thus abolishing the steric hindrance to trigger drug release.^53^^,^^54^^,^^55^

### Electrostatic interaction

Electrostatic repulsion between like-charged particles or attraction between opposite-charged particles is a typical manifestation of electromagnetic force, which could occur between ions, macromolecules, and nanoparticles.^56^^,^^57^ The strength of electrostatic interactions is usually at the same level compared to other types of non-covalent interaction forces and can also occur at long ranges. However, it is notable that a single charged particle may simultaneously interact with multiple charged particles in all directions, and the resultant lack of mutual selectivity may potentially compromise the long-range uniformity of self-assembled nanostructures. A commonly used approach to circumvent this issue is to introduce other more specific regulatory forces to direct the expansion of the supramolecular architecture. For instance, Yue et al. reported a novel multivalent strategy to prepare a free-standing single-layer 2D framework through the self-assembly of positively charged α-cyclodextrin (CD)-based pseudorotaxane as trusses and negatively charged PW_11_VO_40_^4−^ (PWV^4−^) clusters as connection nodes. The four anionic arms of PWV^4−^ nodes could complex with the cationic psudorotaxane for crosslinking via electrostatic attraction, while the treaded CD rings may further guide the 2D arrangement of the pseudorotaxane around the PWV^4−^ nodes through steric effects and lateral hydrogen bonding, leading to the formation of large-scale single-layer membranes with ordered porous structures. The generated pores have a uniform diameter of around 3.4–4.1 nm, which could be readily used for the size-dependent separation of semiconductor quantum dots with an accuracy of around 0.1 nm via simple filtration procedures under reduced pressure.^58^

Electrostatic self-assembly shows evident advantages for the delivery of charged therapeutic substances such as polyelectrolytes, nucleotides, and metal ions. For instance, layer-by-layer self-assembly is a well-tested technology for the functionalization of medical implants, which is realized by the alternating absorption of oppositely charged polyions on engineered surfaces and could be used to create functional coating with composite structures.^59^^,^^60^^,^^61^^,^^62^^,^^63^ Another example in this context is the application of polymer-gene complexes for genetic engineering purposes. Typically, polycations such as polyethylenimine have already been commonly used for the delivery of siRNAs to designated cellular targets, of which the underlying mechanism is that the abundant positively charged amine groups in PEI could complex with the negatively charged phosphate groups in siRNAs through electrostatic interaction, thus enhancing the stability and uptake efficiency of siRNAs by target cells.^64^^,^^65^^,^^66^^,^^67^ In addition, electrostatic complexation between polyions and metal ions is an interesting concept that has drawn increasing interest for tumor therapy.^68^^,^^69^^,^^70^^,^^71^^,^^72^ The electrostatic interaction between charged polymers and metal ions not only enables the synthesis of supramolecular nanomaterials with complex and tailorable topology,^73^ but also maintains the biological reactivity of the metal species to introduce novel therapeutic functions such as biocatalytic therapy, immunostimulation, etc.

### π−π stacking

It is well-established that π electrons in aromatic systems are in a delocalized state. Consequently, when two neighboring aromatic systems are parallel to each other, the dispersion and electrostatic interaction between the π electron systems would generate an attractive non-covalent force to bind the aromatic rings together.^74^^,^^75^^,^^76^ Considering the universal presence of aromatic moieties in various biomacromolecules, π−π stacking is a common phenomenon in nature and involved in various biological events. It is of interest to note that many small-molecule anticancer drugs contain abundant aromatic rings, substantiating the potential application of π−π stacking-based self-assembly strategies for drug delivery applications.^77^^,^^78^ For instance, 2D nanomaterials with delocalized electrons present adequate binding affinity with aromatic ring-containing anticancer drugs through π−π stacking, and their drug loading potential is further amplified by the large surface area, making them promising delivery platforms for a broad spectrum of hydrophobic anticancer drugs.^79^ Typically, in the pioneering study by Sun et al. in 2008, the authors successfully prepared single-layer graphene oxide nanosheets with an average lateral width of around 10 nm and used them for the loading of doxorubicin (DOX), an anthracyline that has been approved as the standard first-line treatment for multiple types of sarcomas. π−π stacking-dependent DOX loading was successfully realized through simple mixing with a high loading ratio.^80^ Similar drug delivery mechanisms have also been validated using other 2D nanomaterials such as black phosphorus,^81^ MoS_2_,^82^ and WS_2_.^83^

## Modulating self-assembly states for stimuli-responsive drug delivery

The intriguing physical and chemical characteristics of supramolecular self-assembly are of immense interest for stimuli-responsive drug delivery to tumor cells. Remarkably, recent insights in this area reveal that the complexation strength of the previously listed non-covalent interactions could be facilely manipulated using specific exogenous and endogenous signals, substantiating the intrinsic advantages of supramolecular self-assemblies for the precise regulation of nanomedicine pharmacokinetics. In this section, we will discuss the nanomaterial strategies based on supramolecular self-assembly principles for tumor-targeted stimuli-responsive drug delivery, which are categorized according to non-covalent interaction modes and types of triggering signals.

### Hydrophobic interaction-based delivery systems

**Figure 1:**
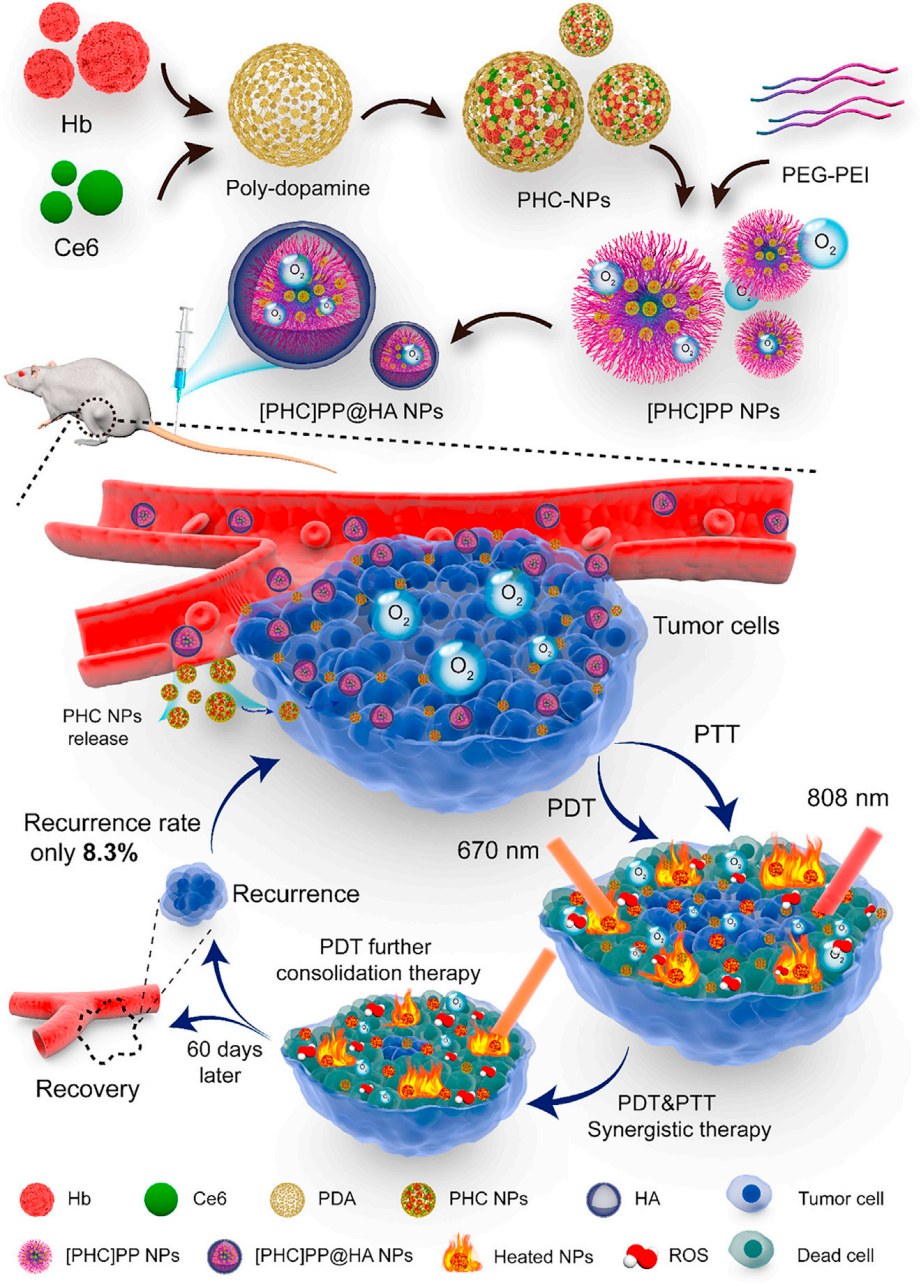
Schematic illustration of the self-assembly procedures for the acidity-responsive size-changeable composite micelle/nanoparticle system and its synergistic PDT/PTT therapeutic mechanism against cancer cells Reproduced with permission from Ref. ^93^ Copyright © 2020, American Chemical Society.

Self-assemblies formed through hydrophobic interaction are highly sensitive to the changes in solvent structure and hydrophobic/hydrophilic balance of the molecular building blocks, both of which would profoundly disrupt the equilibrium state of the supramolecular nanosystems and trigger the release of encapsulated contents. Based on this rationale, scientists have developed a plethora of activatable molecular building blocks by connecting hydrophobic and hydrophilic segments via bioresponsive linkers.^84^ From a general perspective, these linkers could be readily cleaved in response to specific signals in the pathological tumor microenvironment or tumor intracellular compartment, thus disrupting the hydrophobic interaction and initiating drug release. Typically, tumor cells frequently demonstrate multiple biochemical alterations such as higher ROS production, acidic tumor microenvironment, and GSH levels, which distinguish them from normal cells and are commonly used as triggering signals to initiate the transformation of the nanoassemblies.^85^^,^^86^^,^^87^ In the study by Chiang et al., the authors alternatively co-polymerized ROS-sensitive diethyl sulfide and GSH-sensitive cystamine units onto methoxy poly(ethylene glycol) (mPEG) to obtain mPEG-b-P(Des-alt-Cys) copolymer.^88^ Due to the hydrophilicity of the mPEG and hydrophobicity of the P(Des-alt-Cys) segments, the mPEG-b-P(Des-alt-Cys) copolymer could readily self-assemble into aqueously stable micelles after dissolution, and the hydrophobic P(Des-alt-Cys)-constituting core further allows the incorporation of anticancer drug camptothecin. Upon entering tumor cells, the high cytosolic ROS and GSH levels would rapidly decompose the hydrophobic P(Des-alt-Cys) cores and destabilize the micellar structure by disrupting the hydrophobic/hydrophilic balance, leading to complete camptothecin release in tumor cells. Similarly, Wang et al. developed an acidity-responsive size-changeable composite micelle/nanoparticle system for the treatment of hypoxic tumors. It is well established that tumor stroma is a complex ecosystem with high interstitial fluid pressure that severely limits the penetration of extravasated nanoparticles.^89^^,^^90^^,^^91^^,^^92^ Small nanoparticles sized below 10 nm have superior diffusive ability and are thus more capable of penetrating the interior of solid tumors. However, the decreasing nanoparticle size also enhanced the risk of premature clearance from the body after systemic administration. To solve this dilemma, the authors first prepared ultrasmall polydopamine nanoparticles with an average size of around 10 nm for loading hemoglobin and chlorin e6 photosensitizers (PHCs), followed by the encapsulation with a self-assembled micellar structure constituted by benzoic-imine-linked PEI-PEG copolymers and eventually modified with hyaluronic acid (HA) for targeting CD44^+^ tumor cells, and the final nanoproduct has an average diameter of around 140 nm to ensure adequate blood circulation stability. The acidity in TME would trigger the cleavage of the benzoic-imine bond and disrupt the hydrophilic/hydrophobic balance of the micellar shell, thus releasing the small PHCs to enhance their penetration into the hypoxic tumor stroma (Figure 1).^93^ Yang et al. developed a peptidic precursor that could be truncated by mitochondrial-specific sirtuin 5 (SIRT5) enzymes to remove the hydrophilic tail and initiate hydrophobic interaction of the residual segments, thus restoring their fluorescence capability for mitochondrial imaging in living cells.^94^ Another group reported a cyclic peptide-bridged amphiphilic diblock copolymer that could self-assemble into cylindrical micelles driven by hydrophobic interaction and hydrogen bonding for light-controlled DOX-delivery.^95^ These studies collectively supported the concept of using endogenous biochemical cues to alter the hydrophobicity of structural components to trigger drug release from self-assembled nanostructures. However, a major issue concerning the translation of these endogenous trigger-responsive systems is their applicability in the complex and highly heterogeneous ecosystem in real-life patients. For instance, many inflammatory sites may also present acidic microenvironmental pH and cause off-site activation of the pH-responsive drug delivery systems, leading to undesirable side effects. On the other hand, the difference of a certain biochemical cue between normal and tumor tissues is sometimes not significant enough to potentiate precision therapy. These issues may impede the clinical application of endogenous trigger-responsive nanotherapeutics for cancer treatment and put up further challenges to optimize the supramolecular self-assembly designs.

**Figure 2:**
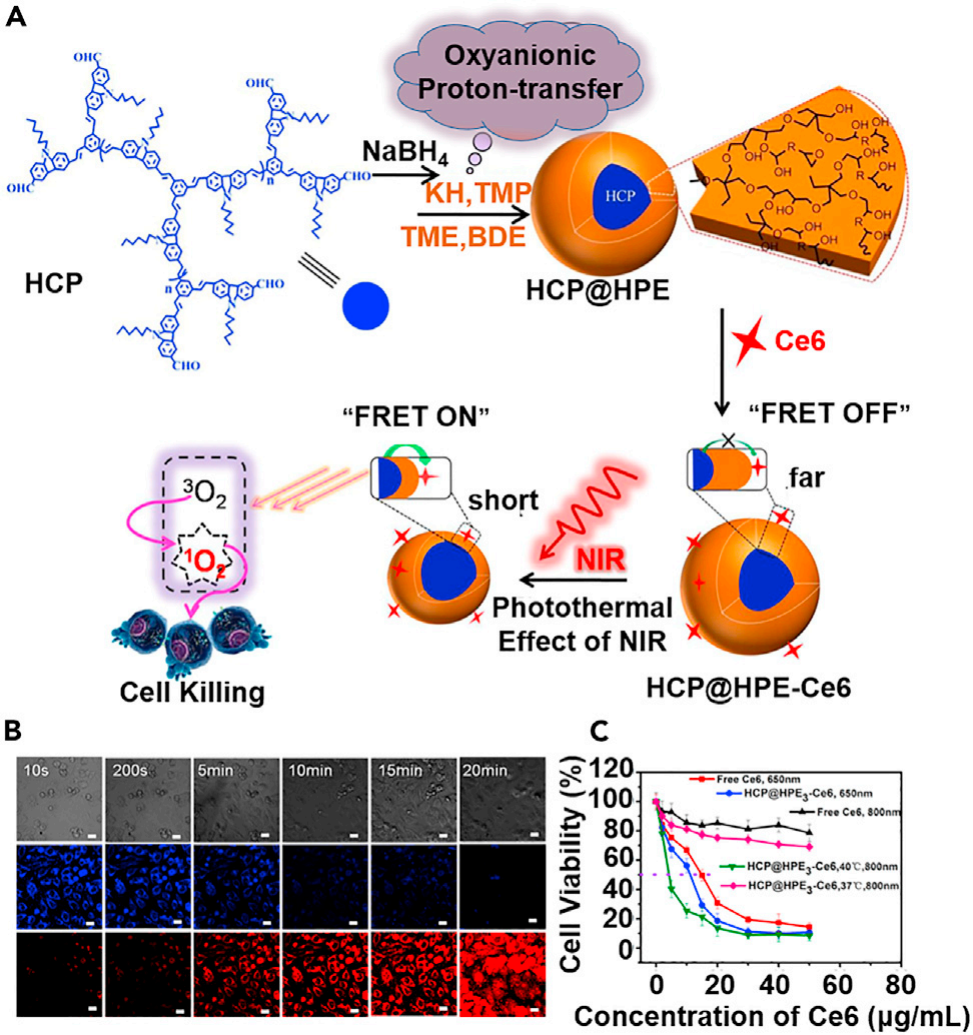
Hydrophobic interaction-dependent HCP@HPE self-asselblies for NIR-triggered two-photon-FRET PDT (A) Schematic illustration of hydrophobic interaction-driven self-assembly of the HCP@HPE micelles and the photothermal-dependent LCST transformation of HPE for activating two-photon-FRET PDT. (B) Two-photon laser scanning fluorescence microscopic analysis of micelle-treated tumor cells, revealing the gradual activation of the two-photon-FRET process under NIT laser illumination. (C) NIR-dependent photothermal effect boosts the cytotoxicity of the HCP@HPE against tumor cells. Results are shown as mean± standard error. Reproduced with permission from Ref. ^107^ Copyright © 2016, American Chemical Society.

Alternatively, there is ample evidence that external signals such as light and heat could be used to regulate the hydrophobicity of chemically engineered polymers and control drug release. A prominent example in this context is the nanostructures constituted by polymers with upper/lower critical solution temperature (UCST/LCST) behavior.^96^^,^^97^^,^^98^ For typical LCST polymers, they are generally hydrophilic when the temperature is below LCST. However, they would undergo a phase separation process as temperature increases and become increasingly hydrophobic. Contrastingly, UCST polymers show a reversed trend that they will switch from a hydrophobic state to hydrophilic in response to temperature increases. The most extensively studied example of these thermoresponsive polymers is poly(N-isopropylacrylamide) (PNIPAM), which has attracted broad interest for drug delivery applications considering that its LCST is around 33°C and near normal body temperature. The LCST polymer-based thermoresponsive drug delivery strategy is excellently demonstrated in the report by Wei et al. in 2006, in which the authors synthesized poly(N-isopropylacrylamide-b-methyl methacrylate) block polymers (PNIPAAm-b-PMMA) for the delivery of a model drug prednisone acetate.^99^ Owing to the hydrophilicity of PNIPAM and hydrophobicity of PMMA segments, the PNIPAAm-b-PMMA polymer could readily self-assemble into micelles in an aqueous environment when the temperature is below LCST, while the inner core allows the incorporation of lipophilic drugs through hydrophobic interaction. However, upon external heating, the PNIPAM segments would become hydrophobic and cause the total collapse of the micellar structure, leading to the burst release of encapsulated drugs. Nevertheless, previous insights reveal that PNIPAM may induce significant acrylamide-associated cytotoxicity and neurotoxicity, and several alternative LCST polymers have been developed for translational purposes,^100^ including poly(N-vinylpiperidone),^101^ poly[oligo(ethylene glycol)-methacrylate],^102^ poly(N-dimethylacrylamide),^103^ elastin side-chain polymer,^104^ polycaprolactone,^105^ hyperbranched polyether,^106^ etc. For instance, Huang et al. developed a core-shell micelle to enhance the controllability and efficacy of photodynamic therapy by exploiting the LCST behavior. The micelle system comprised a two-photon absorbing hyperbranched conjugated polymer core (HCP) and a thermoresponsive hyperbranched polyether shell (HPE), while Ce6 photosensitizers were conjugated onto the outer surface.^107^ Under NIR illumination, the HCP content could absorb the incident light and convert it into thermal energy for efficient photothermal heating, which would subsequently trigger the collapsing of the HPE shell and pull the Ce6 molecules closer to the HCP compartment, thus enabling two-photon-activated fluorescence resonance energy transfer from HCP to Ce6 to produce abundant singlet oxygen for tumor inhibition (Figure 2). Alternatively, there are also attempts to exploit UCST polymers for thermoresponsive drug delivery. Based on the UCST property of acrylonitrile-acrylamide hybrid copolymers, Li et al. prepared poly(AAm-co-AN)-g-PEG copolymer through procedural solution polymerization.^108^ The poly(AAm-co-AN) segment has high hydrophobicity when the temperature is below 43°C, which could balance the hydrophilicity of PEG segments to form DOX-loaded micellar structures sized around 50 nm. No significant drug release was observed when the temperature was below 43°C, supporting the aqueous stability of generated micelles. However, when localized heating was applied to raise the temperature above 43°C, the poly(AAm-co-AN) segment would undergo an immediate hydrophobic-to-hydrophilic transition and cause the complete dissolution of the micelles, leading to a sharp increase in DOX release for remotely controllable tumor therapy. Nevertheless, most of the thermo-responsive polymers in current reports are non-degradable, which may lead to potential short-term and long-term health risks after systemic or localized administration. On the other hand, hybridizing LCST or UCST polymers with degradable linkers may improve their biocompatibility but also affect their thermo-responsive behaviors, thus requiring new synthetic technologies to achieve balanced drug delivery performance and *in vivo* safety.

### Hydrogen bonding-based delivery systems

**Figure 3:**
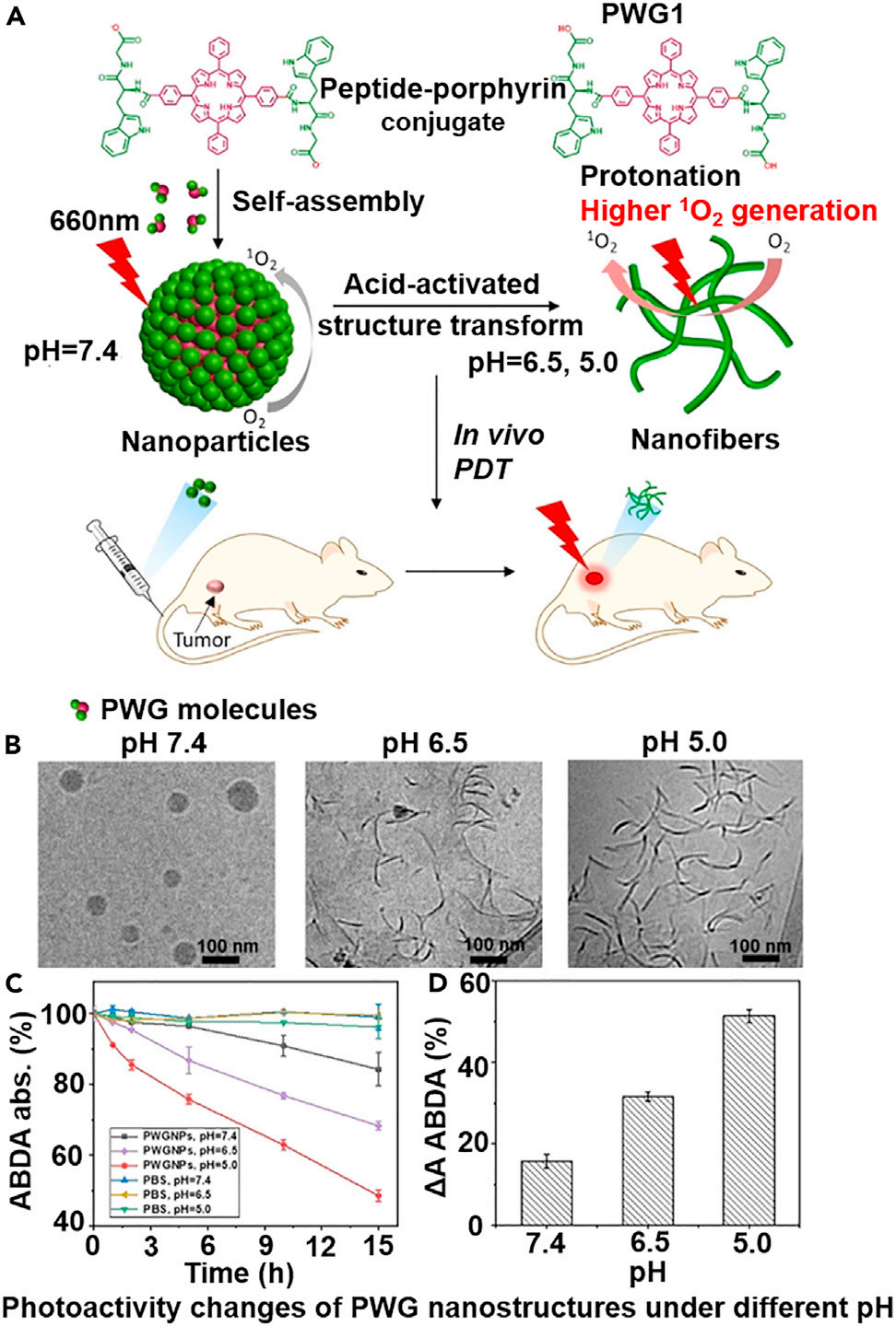
Construction and tumor-responsive activation of peptide-porphyrin self-assemblies for enhanced PDT (A) Molecular structure of the acid-responsive peptide-porphyrin building blocks and schematic illustrations of their hydrogen bonding-mediated transformation from spherical shapes to nanofibers. (B) TEM images showing the morphological transformation process of the self-assembled nanostructures from pH 7.4, 6.5, and 5.0 from left to right. (C) Time-dependent analysis on the ROS production of the self-assembled PWG nanostructure under different pH conditions. Results are shown as mean± standard error. (D) Comparative analysis on the ROS-generating capacity of PWG nanostructures under different pH conditions. Results are shown as mean ± standard error. Reproduced with permission from Ref. ^111^ © 2020 The Authors. Published by Wiley-VCH GmbH.

Extending from the previously mentioned factors that govern the strength of hydrogen bonding in self-assembled nanostructures, several strategies have been recently proposed to dynamically regulate the hydrogen bonding patterns for realizing stimuli-responsive drug release, which primarily focus on the modulation of molecular configurations to break or form hydrogen bonding. Typically, Liu et al. developed morphologically transformable micelles with high tumor retention for chemo-photodynamic therapy, which is constructed through the polyvalent self-assembly of heterogeneously functionalized linear triblock copolymers.^109^ The copolymers comprised a hydrophobic head (Ce6 photosensitizers or bilirubin [BR] anticancer drug), a middle peptidic segment with hydrogen bonding potential, and a hydrophilic terminal PEG segment. The mixture of the heterogeneously modified copolymers could self-assemble into micelles after dissolution in aqueous environment through the hydrophobic interaction of Ce6/BR units. Interestingly, upon laser irradiation at 650 nm at the tumor regions, the photodynamically generated ROS would rapidly detach the BR moieties and disrupt the total hydrophilic/hydrophobic balance of the micellar system. However, due to the hydrogen bonding between the intermediate peptidic segment, the self-assembled micelles would not dissociate into single molecule but transform into fiber-like shapes, which could substantially enhance both the retention and penetration of Ce6 molecules in solid tumors for enhanced photodynamic efficacy. A similar hydrogen bonding-dependent morphological transformation design was reported by Cheng et al., aiming to improve tumor-specific retention and penetration of anticancer agents for enhanced chemo-photodynamic therapy.^110^ The self-assembled nanosystem comprised three major components including a pentapeptide FF-AmpF-FF (AmpF) and two AmpF-derivatives which were individually conjugated with camptothecin anticancer drug or new indocyanine green IR820 photosensitizers. Owing to the strong hydrogen bonding between the peptides, they could form quadruple helices in aqueous environment of neutral pH (7.4). However, after reaching the mildly acidic TME (pH: 6.5), the strength of hydrogen bonding decreased significantly, while the hydrophobic interaction and π-π stacking between the peptides became the dominant force, which would drive the morphological transformation from linear superhelices to spherical nanoparticles sized around 90 nm, leading to the exposure of the peptides on nanoparticle surface while therapeutic substances were stored in the inner compartment to improve tumor penetration. It is important to note that the pH-triggered nanoparticle transformation was completely reversible, and the spherical nanoparticles would switch back to the helical shape after escaping from tumor lysosomes to the cytosol compartment, thus enhancing the tumor cell retention of the therapeutic agents. Based on similar pH-regulated intermolecular hydrogen bonding mechanisms, Sun et al. reported a morphologically transformable tryptophan-glycine peptide-porphyrin nanoparticle for photodynamic therapy, which showed normal spherical shapes under neutral pH but may transform into fiber-like shapes when exposed to acidic TME.^111^ This transformation process was driven by the protonation of tryptophan-glycine peptide under acidic conditions, which would strengthen the intermolecular hydrogen bond and trigger structural rearrangement. The sphere-to-fiber transformation could enhance intersystem crossing to boost the yield of singlet oxygen under laser stimulation, as well as impede the diffusion of the nanomaterials back to the bloodstream to enhance tumor accumulation (Figure 3). Tang et al. reported that tetrakis(4-amidiniumphenyl)methane and azobenzenedicarboxylate could form hydrogen-bonded organic nanoframeworks for enzyme delivery, which could maintain the biocatalytic activity of enzymes during blood transportation while becoming dissociated in endosomes to release the enzymes for therapeutic purpose.^112^Furthermore, contrary to the pH-induced dissociable nanosystems described above, An et al. reported an *in vivo* self-assembly drug depot system that could be activated by the X-linked inhibitor of apoptosis protein in tumor cells, which could eventually self-assemble into fiber-like superstructures under hydrogen bonding directed growth to achieve greater tumor retention.^113^ Notably, despite the promise of these morphologically tunable self-assembled nanosystems for overcoming the obstacles in drug delivery to solid tumors, the size/shape transformation and cellular uptake of these nanosystems are happening at the same time and it is important to consider the kinetic profiles to the transformation process under specific stimulus to improve their tumor-targeted uptake before clearance.

### Host-guest interaction-based delivery systems

**Figure 4:**
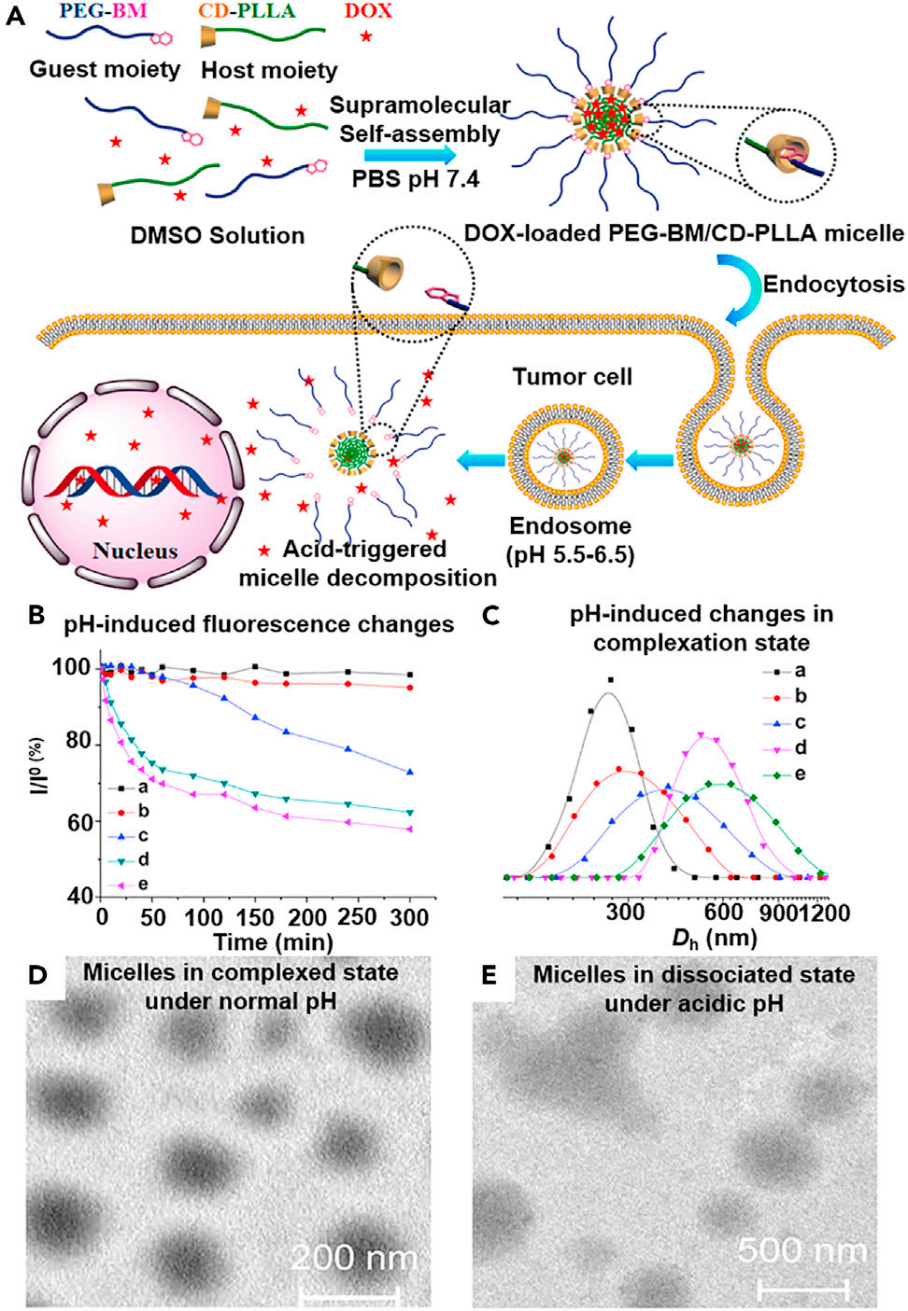
pH-triggered dissembly of host-guest micelles for tumor-responsive DOX release (A) Schematic illustration of the host-guest interaction-mediated self-assembly of PEG-BM/CD-PLLA micelles and their acidity-triggered degradation in tumor endo/lysosomes. (B/C) Time-dependent release of model drug and size changes of PEG-BM/CD-PLLA micelles under graded pH values via fluorescence spectroscopy. (A) pH 7.4, (B) pH 6.8, (C) pH 6.5, (D) pH 6.0, (e) pH 5.5. (D/E) TEM analysis on the pH-triggered disassembly of PEG-BM/CD-PLLA micelles from pH 7.4 to pH 5.5. Reproduced from Ref. ^117^ Copyright © 2015, American Chemical Society.

Inspired by the dynamic and reversible nature of host-guest interaction, many strategies have been developed to realize on-demand drug delivery to tumors, and the triggering signals encompass a wide range of physical and biochemical cues. The most common examples of macrocyclic moieties for biomedical applications include cyclodextrins, calix[n]arenes, pillar[n]arenes, and cucurbit[n]urils. In the context of drug-macrocyclic ring host-guest complexes, previous studies reveal several factors that may accelerate their dissociation rate. For instance, increasing environmental acidity could potentially weaken the hydrogen bonding between the macrocyclic ring and guest molecules, while elevating temperature could enhance the Brownian motion of included guest molecules and facilitate their escape from the entrapped state.^114^^,^^115^ On the other hand, the design of a nanovalve-controlled stimuli-responsive drug delivery system is more complex and requires additional engineering at molecular and nanoscale levels. Based on the capping strategy and the underlying supramolecular principles, current nanovalves designs could be categorized into two fundamental types, which are (1) dissociable nanovalves, where nanovalves are capped onto the surface of nanocarriers via cleavable linkers and (2) non-dissociable but moveable nanovalves, which could change their distance from the nanocarrier surface and switch between an on-off state. A typical example in the context of the first strategy was reported by our group in 2013. In this study, we successfully immobilized disulfide-containing tetraethylene glycol molecules on the surface of doxorubicin-loaded hollow mesoporous silica nanoparticles as the anchoring structure for treading α-cyclodextrin (α-CD) through host-guest inclusion, followed by the end-capping with folic acid-containing ligands for α-CD immobilization.^116^ The α-CD rings could adequately seal the pore openings to prevent the premature leakage of DOX molecules from the hollow mesoporous silica nanosubstrates, while the folic acid end groups may confer targeting specificity against tumor cells overexpressed with folate receptors. After being taken in by tumor cells, the abnormally upregulated GSH levels would readily cleave the disulfide bond and remove the whole rotaxane capping moiety from the nanoparticle surface, thus triggering the DOX-dependent anticancer therapy. Alternatively, Zhang et al. reported a pH-responsive micellar system for triggerable DOX release in tumor cells, which was fabricated through the host-guest interaction-driven self-assembly of benzimidazole-terminated poly(ethylene glycol) (PEG-BM) and β-cyclodextrin-modified poly(l-lactide) (CD-PLLA).^117^ The micelle could be readily obtained in a one-pot approach by mixing PEG-BM, CD-PLLA, and DOX in aqueous media at neutral pH (7.4), during which the hydrophobic CD-PLLA molecules would first self-aggregate into a nanocore for DOX encapsulation, followed by the insertion of BM moiety into the cavity of β-CD for PEG-BM modification, leading to the formation of a hydrophilic anti-opsonization corona around the hydrophobic cores to enhance their *in vivo* stability and passive tumor homing effect. After entering the acidic tumor lysosomes, the local high acidity (pH 5.5–6.5) would weaken the host-guest interaction between PEG-BM and CD-PLLA to shed the PEG shell and trigger DOX release (Figure 4). Still, it must be recognized that the actual biological environment in real-life patients is a highly complex system with numerous biomolecules of diverse shapes, which may competitively bind with the host/guest moieties and cause the premature activation of the nanosystems, thus increasing the risk of non-specific toxicity. Consequently, it is important to comprehensively investigate the interaction between host-guest interaction-based delivery systems and biological environment under clinical models.

**Figure 5:**
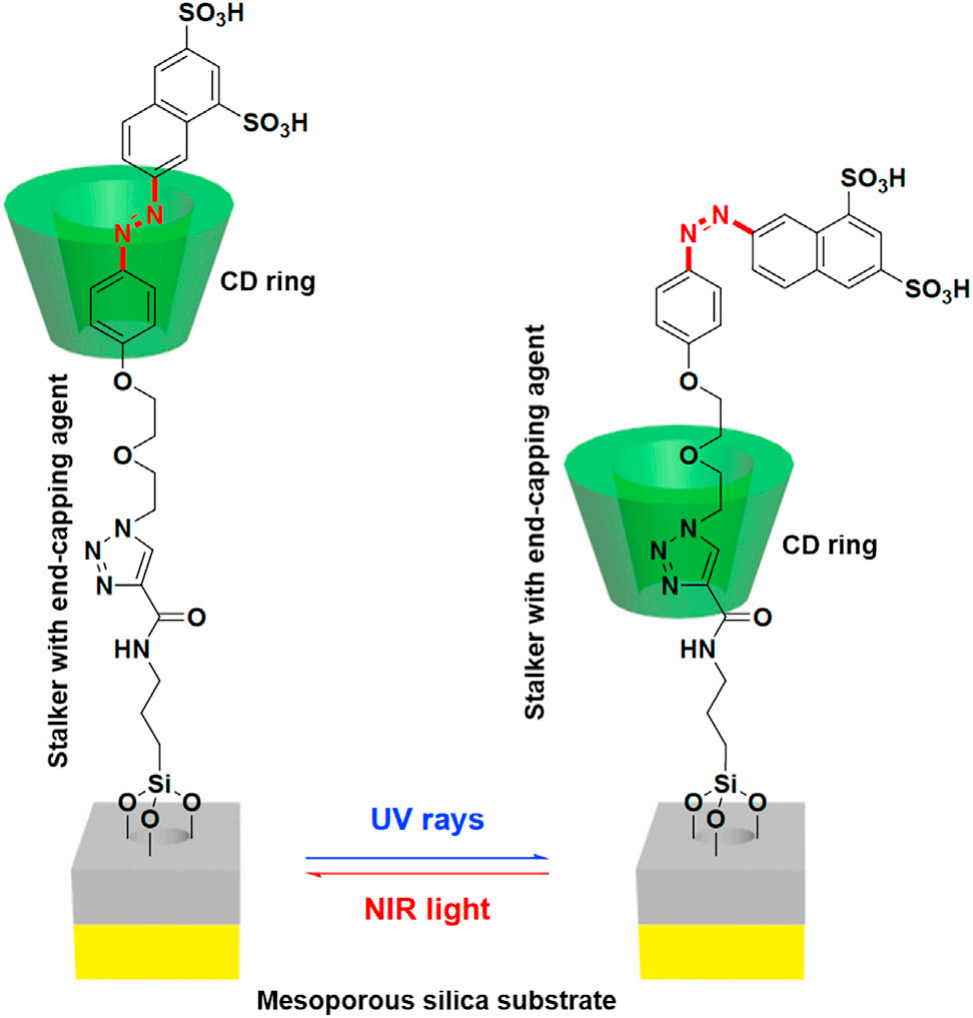
Mechanism for the NIR-regulated on-off switch of α-CD-disulfonated azobenzene rotaxane on mesoporous silica-coated gold nanorods Reproduced with permission from Ref. ^118^ © ROYAL SOCIETY OF CHEMISTRY.

Previous studies on the molecular characteristics of rotaxane revealed that the movement of the macrocyclic ring along the treaded axis could be facilely regulated via steric hindrance, thus allowing the facile control of the distance between the macrocyclic ring to nanoparticle surface for enabling the on-off reversible switch of drug release. In 2013, our group developed a rotaxane system based on α-CD and disulfonated azobenzene, which was used for the surface modification of mesoporous silica-coated gold nanorods for realizing remotely controllable DOX release (Figure 5).^118^ The α-CD/azobenzene rotaxane is permanently conjugated onto the silica surface through a copper-catalyzed click reaction. Under normal situations, the azobenzene moiety remained in a *cis* state that pushed the α-CD ring closer to the silica surface, thus blocking the diffusion of encapsulated DOX molecules. However, under NIR laser stimulation (808 nm), the gold nanorods could rapidly elevate ambient temperature through photothermal heating and cause the *cis*-to-*trans* isomerization of the azobenzene moiety through thermal relaxation. Consequently, the α-CD ring could freely move along the molecular axis and unblock the pores to trigger drug release, leading to precise on-off drug release patterns. A different drug delivery strategy based on the azobenzene-enabled light-controlled on-off switch of nanovalves was also reported by Tarn et al., in which the authors synthesized two types of α-CD/azobenzene rotaxanes with different stalk lengths for regulating the release of model drug molecules of graded sizes from silica nanochannels, where the *trans*-to-*cis* isomerization of the azobenzene moiety under light irritation would cause the α-CD ring to move to the far end of the molecular axis and unblock the pores in MSNs to initiate drug release, while removing the light source would reset the complexation state of rotaxane and seal the pore opening.^119^ The authors revealed that the length of the stalk is a key parameter determining whether a drug molecule of specific size could escape from the carrier substrates, as the results identified that only alizarin red S (length: 1.2 nm) molecules could be loaded into and released from rotaxane-functionalized MSNs with a stalker length of 1.6 nm, while propidium iodine (length: 1.5 nm) and Hoechst 33,258 (length: 2 nm) cannot be loaded into the nanochannels despite prolonged incubation. In contrast, modifying MSN using α-CD/azobenzene rotaxanes with a stalk length of 2.8 nm allowed the loading of molecules smaller than 2 nm and showed light-regulated release control over alizarin red S and propidium iodine, but Hoechst 33,258 still remained excluded.

The reversible host-guest interaction mechanism was also employed to develop non-nanovalve drug delivery systems with intricate tumor responsiveness. Yang et al. heterogeneously modified the two ends of WYFK peptides with mitochondrion-targeting TPP moiety and ferrocenyl group. The ferrocenyl groups could act as guest moiety to be inserted into pillar[6]arene to form a stable host-guest complex, which would further self-assemble into nanospheres exposing the pillar[6]arene units through the hydrogen bonding and π-π interaction between the WYF segments. The acidic lysosome would trigger the detachment of pillar[6]arene units and cause the reassembly of the remaining Fc-WYFK-TPP units to form nanospheres exposing TPP moieties for mitochondrial targeting, eventually leading to efficient ferroptotic tumor cell death.^120^ Based on similar host-guest recognition mechanisms, the same group of authors also incorporated boron dipyrromethene (BODIPY)-bearing methylimidazolium into pillar[6]arene to form photothermal-capable complexes, which could further self-assemble into nanovesicles for cisplatin encapsulation. Light irritation after uptake by tumor cells would trigger the dissociation of the functional host-guest complex and release the payload in a responsive manner.^121^ Feng et al. developed a tumor-responsive PDT nanosystem through the self-assembly of pyridinium-functionalized tetraphenylethylene-*p*-sulfonatocalix[4]arene host-guest complex. Due to the calixarene-mediated restriction of intramolecular motion, the supramolecular nanosystem showed negligible phototoxicity under a dark environment, while the addition of 4,4′-benzidine dihydrochloride would compete with the tetraphenylethylene-based photosensitizer and displace them from the calixarene cavity, thus restoring their PDT activity for on-demand tumor therapy.^122^ Sun et al. synthesized supramolecular peptide prodrug through the host-guest-interaction between cucurbit[7]uril and Phe moieties on Phe-Phe-Val-Leu-Lys-camptothecin conjugates, which could further self-assemble into spherical nanoparticles. After uptake by cancer cells, the overexpressed spermine would complete with the peptidic CPT prodrug and displace them from the cucurbituril cavity to trigger drug release.^123^

### Electrostatic interaction-based delivery systems

**Figure 6:**
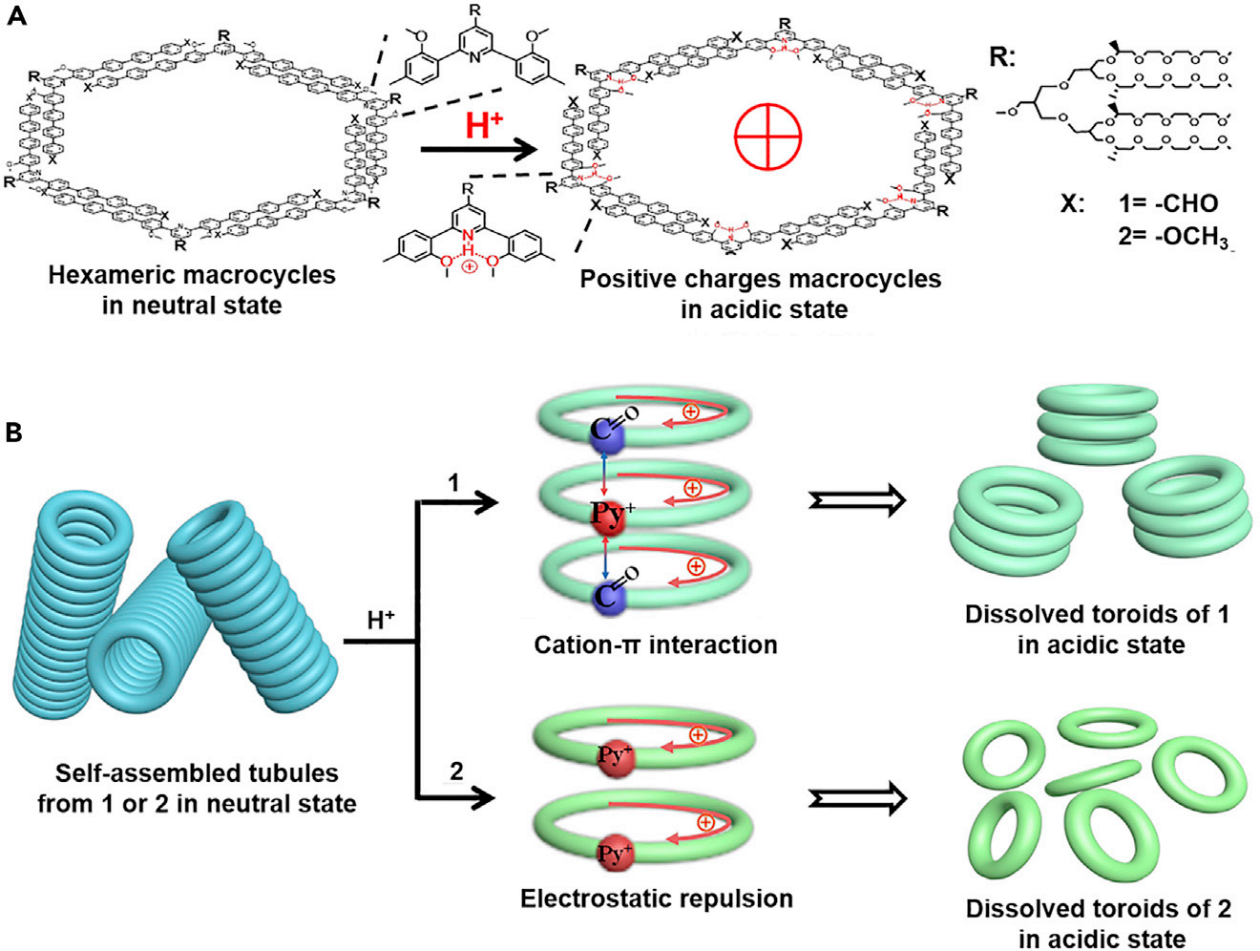
pH-triggered transformation of the macrocycle self-assemblies (A) Mechanism of the pH-regulated charge conversion in macrocycles constructed through the self-assembly of V-shaped aromatic amphiphiles (precursor 1 and 2). (B) Schematic illustration of acidity-mediated dissociation of self-assemblies based on precursor 1 or 2. Dissociation of precursor 1-based self-assembly is driven by cation-π interaction, while that for precursor 2 is driven by electrostatic repulsion. Reproduced with permission from Ref. ^124^ © 2019 The Author(s).

The construction of electrostatic self-assemblies predominantly relies on the attraction force between oppositely charged entities, which is crucial for maintaining the supramolecular architecture and stabilizing therapeutic agents. Therefore, the central idea to realize stimuli-responsive drug release from electrostatic self-assembled nanocarriers is to induce charge reversal of specific components including certain structural elements or the loaded drugs. For instance, in a recent study by Huang et al., the authors reported a pH-sensitive nanoassembly of ethylene oxide-modified aromatic macrocycles for the acidity-triggerable delivery of DOX to tumor cells (Figure 6).^124^ The conjugation of ethylene oxide segments could substantially improve the aqueous solubility of the aromatic units, which would further form tubular nanostructures through cation-π interaction. Meanwhile, the amphiphilic nature of the macrocycles also allowed the efficient loading of hydrophobic DOX molecules. Under acidic conditions, the inner surface would become protonated, and the increasing electrostatic repulsion would trigger the complete decomposition of the tubular nanostructure while detaching DOX molecules. In another study by Han et al., the authors prepared electrostatic multilayered self-assembly on the surface of DOX-loaded TAT peptide-modified MSNs.^125^ Due to the strong positive charge of the TAT peptides, they were used as the base layer for the absorption of negatively charged poly(allylamine hydrochloride)-citraconic anhydride (PAH-Cit), followed by an additional cationic galactose-modified trimethyl chitosan-cysteine (GTC) layer for the incorporation of anionic siRNAs. Due to the strong electrostatic attraction between individual layers, the multilayer-stabilized MSNs remained stable during blood circulation and in TME with negligible DOX and siRNA leakage. However, after reaching the acidic tumor lysosomes, the relatively strong acidity (pH 5.0) would protonate the PAH-Cit contents and lead to a charge reversal from negative to positive, and the resultant electrostatic repulsion would immediately dissociate the whole multilayered coating and trigger the release of DOX and siRNAs. Interestingly, the protonation of PAH-Cit would cause a massive proton influx into the tumor lysosomes and facilitate the lysosomal escape of released DNA and siRNA molecules through the so-called proton-sponge effect. Similarly, Cao et al. employed dynamic combinatorial chemistry (DCC) principles to screen the fittest negatively charged octameric disulfide macrocyclic molecule (8mer) by introducing a positively charged template and hydrophobic drug molecules into a dynamic combinatorial library (DCL) from a dithiol building block.^126^ The 8mers could readily co-assemble with the template and DOX through multivalent interactions in an aqueous solution into nanorods with a high DOX loading ratio of around 40% and the lowest energy levels. After translocating to the acidic tumor lysosomes, the amine moieties in DOX molecules and the carboxylic groups in 8mers would become protonated, which would weaken the attraction between 8mer and cationic template while enhancing the electrostatic repulsion between DOX and the template, thus destabilizing the co-assembly to trigger DOX release. Guo et al. co-assembled Polyethylenimine-modified silica (SiO_2_–PEI) NPs and zwitterionic fluorescent carbon dots (CDs) onto the surface of emulsion droplets, where the SiO_2_–PEI NPs and CDs could form a stable shell through strong electrostatic attraction in between but rapidly become disintegrated under tumor-relevant acidic conditions.^127^ Li et al. simultaneously loaded chlorin e6 (Ce6) and rose bengal (RB) inside positively charged porous UiO-68-NH_2_ MOF and further complexed Nd^3+^-sensitized UCNPs onto their surface via electrostatic interaction, thus enabling efficient PDT under NIR light irritation.^128^ Nevertheless, it should be mentioned that the pH-driven dissociation of electrostatic complexes is not a threshold event, as the degradation rate is positively correlated with the environmental acidity. Consequently, electrostatic complexes are more prone to premature drug leakage before reaching their eventual cellular targets, warranting additional engineering of the structural components.

### π-π stacking-based delivery systems

**Figure 7:**
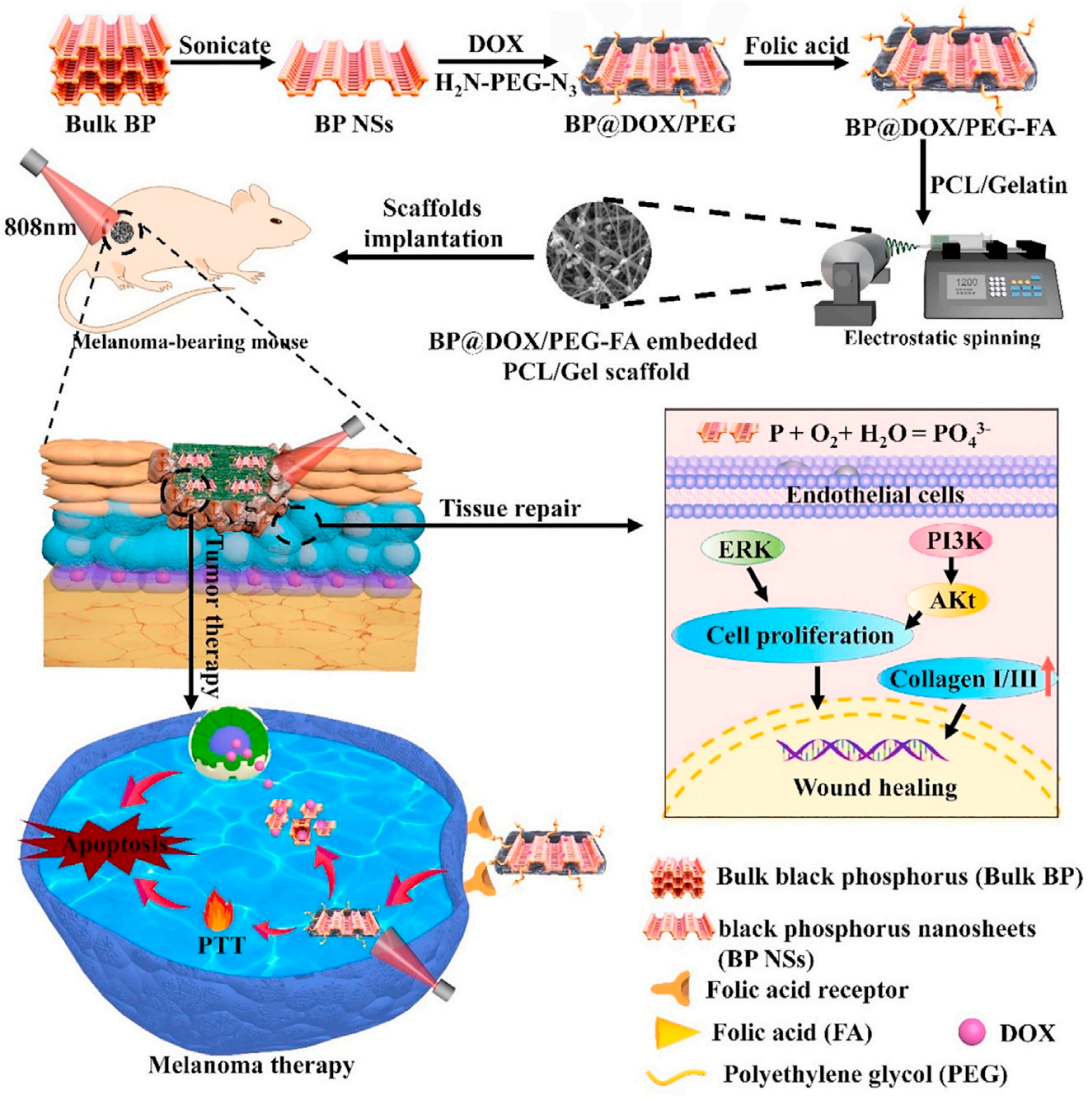
Schematic illustration of DOX-bound BPNSs and their integration into bioresorbable electrospun scaffold for chemotherapy of resected tumors Reproduced with permission from Ref. ^130^ © 2020 Elsevier Ltd. All rights reserved.

Considering the physical and biochemical characteristics of π–π stacking interactions, there are two primary strategies to trigger drug release from π–π stacking-dependent self-assembled nanosystems, which are (1) heating-mediated enhancement of Brownian motion of loaded drugs and (2) introduction of repulsive non-covalent forces. The first strategy is frequently used conjunctionally with photothermal-capable carrier substrates with delocalized π electrons such as graphene oxide and black phosphorus, potentiating on-demand drug release through remotely controllable NIR lasers.^129^ For instance, our group reported in 2021 that DOX molecules could be absorbed onto the surface of folic acid-modified black phosphorus nanosheets (BPNSs) through π–π stacking to form stable complexes, which are further embedded into bioresorbable electrospun gelatin-poly(ε-caprolactone) (GP) scaffolds for the sustained treatment of resected tumors (Figure 7).^130^ Under repetitive NIR laser treatment, the BPNS-mediated photothermal effect caused the sol-gel transition of the GP network and induced the detachment of embedded DOX-BPNS complexes, which were further captured by residual tumor cells through folic acid-mediated targeting effect. The follow-up NIR treatment would further enhance the random Brownian movement of surface-bound DOX molecules and enable their on-demand release for chemotherapy. A similar photothermal-actuated dissociation mechanism has also been employed by many other groups for remotely triggerable chemotherapy. Xie et al. developed BP hydrogels for the π–π stacking-mediated incorporation of emetine, which could be released under NIR-triggered photothermal effect and inhibit stress granule formation in tumor cells, in turn sensitizing them to BP-mediated photothermal therapy.^131^ Wang et al. integrated BP into poly(lactic-co-glycolic acid) nanoshells for the tumor-targeted delivery of docetaxel (DTX), which could be disintegrated under NIR treatment and release DTX into tumor cytosol. ^132^ However, accumulative insights also showed that the potential negative impact of the light treatment cannot be neglected. Typically, tumors are frequently deeply buried within the human body and even NIR light cannot adequately penetrate the covering tissues. Meanwhile, the incident light may induce potential phototoxicity by reacting with biomolecules in the affected area. These obstacles could be overcome with the implementation of more advanced precision light resources such as optical fiber-based devices.

**Figure 8:**
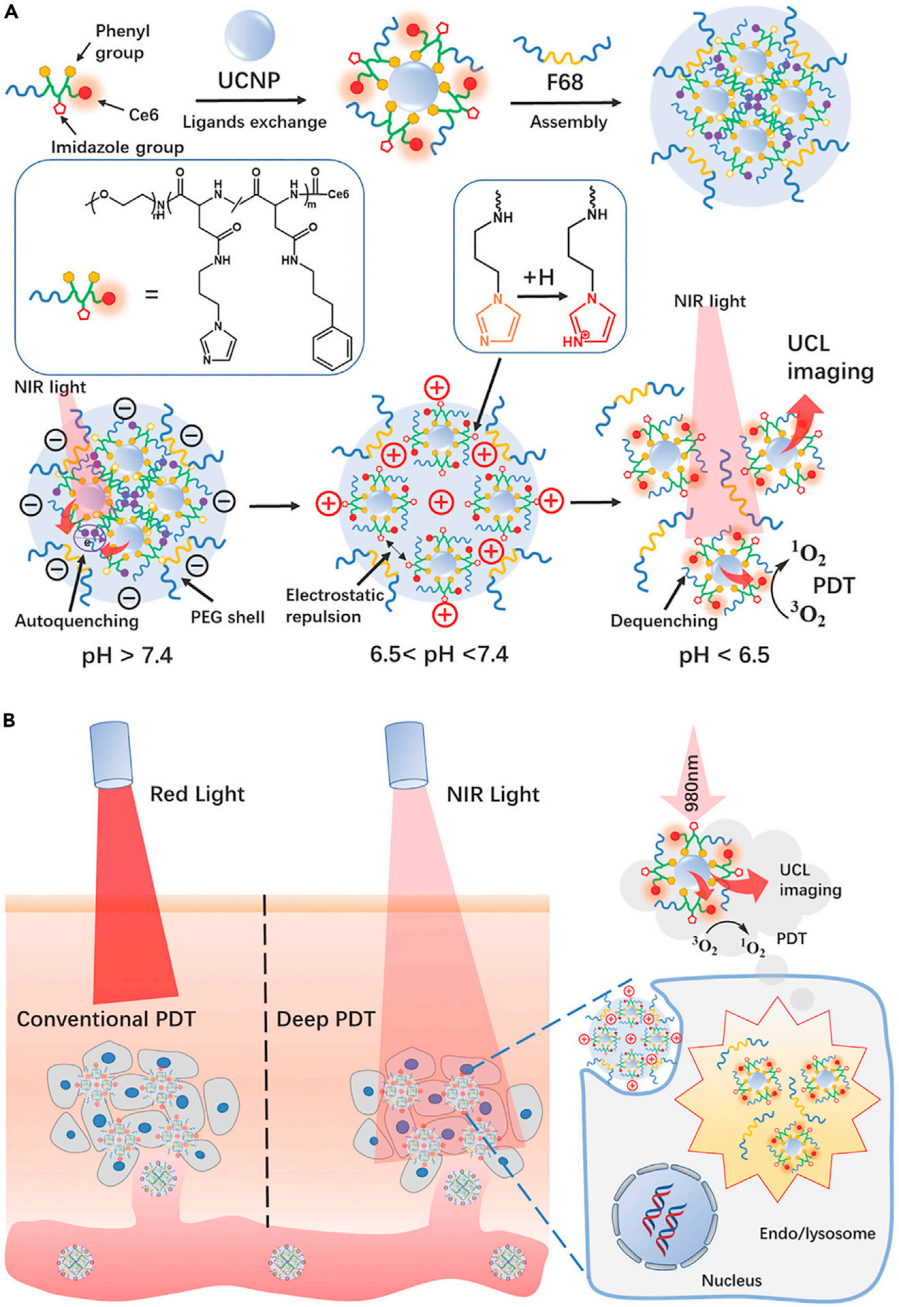
NIR-triggered deep PDT with π–π-stacked functional UCNPs (A) π–π stacking-mediated self-assembly of the polymeric ligand with UCNPs under the assistance of F68 and the pH-induced electrostatic repulsion to trigger disassembly. (B) Therapeutic mechanism of the pH-responsive nanoassembly against solid tumors. Reproduced with permission from Ref. ^133^ © 2018 WILEY-VCH Verlag GmbH & Co. KGaA, Weinheim.

Alternatively, the distinct difference between tumor tissues/cells and their normal counterparts could be exploited to trigger controllable changes in their physical or biochemical characteristics such as molecular geometry and charge status, thus generating additional attractive or repulsive forces to overcome the π–π stacking interactions and trigger drug release. Typically, Li et al. designed a multifunctional polymeric ligand comprising a poly(ethylene glycol)-poly(β-benzyl-*l*-aspartate) backbone with 1-(3-aminopropyl) imidazole (API) and 3-phenyl-1-propylamine (PPA) branches as well as Ce6 terminals for the modification of upconverting nanoparticles (UCNPs), which was realized through the π–π stacking between phenyl branches in the functional copolymer and UCNP surface (Figure 8).^133^ The ligand-modified UCNPs could further self-assemble into negatively charged nanoparticles of around 120 nm under the assistance of Pluronic F68 surfactants. Under normal pH conditions, the nanoparticles remained stable and the Ce6 molecules were in an aggregated state, leading to the self-quenching of their photodynamic activities. However, after reaching the acidic TME, the imidazole side groups would be protonated and become positively charged, and the resultant electrostatic repulsion would cause the large nanoparticles to disintegrate into smaller monodispersive positively charged UCNPs, leading to enhanced tumor penetration and uptake. Furthermore, the repulsion between individual ligands would also reverse the aggregated state of Ce6 molecules and abolish the self-quenching, thus allowing the substantial recovery of their PDT activity. Chen et al. developed a novel method to regulate the morphology of self-assemblies based on protein-drug conjugates (PDCs) through endogenous signal-triggered alteration in intermolecular interactions. The authors first prepared a model PDC comprising DOX and a short KGFRWR peptide, which could co-assemble with PEG molecules heterogeneously functionalized by polyglutamic acid and galactosamine on opposing ends.^134^ The cationic PDCs could form elongated micellar cores in an aqueous environment through π–π stacking, while the anionic functionalized PEG molecules would bind to their surfaces via electrostatic attraction. Interestingly, the absorption of PEG species also blocked the excessive growth of the self-assemblies, eventually leading to the formation of uniform rod-like nanostructures. After reaching the tumor lysosomal environment through galactosamine-mediated targeted uptake, the local acidity would cause the protonation of carboxylic groups in the polyglutamic acid segment and weaken the electrostatic attraction between the PEG shell and PDC-based core to expose the PDCs, while the electrostatic repulsion between the highly positively charged KGFRWR peptides would further cause the disassembly of the micellar structure to potentiate mitochondrial-targeted DOX delivery. Guo et al. used hydrophilic arginine to modify camptothecin (CPT) via hydrolyzable ester bond and the engineered CPT molecules could self-assemble into helical nanofibers through π-π stacking and hydrophobic interactions. In the acidic tumor lysosomes, the ester bond would be cleaved to remove the hydrophilic tail and induce the dissociation of the helical nanofibers to release the CPT drug.^135^ Wang et al. reported that DOX, gossypol, and polydopamine could self-assemble into compact lollipop-like nanoparticles via π-π stacking interaction, while the acidic pH in tumor lysosomes could disrupt the π-π stacking interaction to trigger the release of free DOX and gossypol.^136^

## Concluding remarks and future perspectives

The engineering of self-assembled nanomedicine through non-covalent interactions has attracted immense interest for the pharmaceutical development of novel anticancer agents. Particularly, their supramolecular molecular cooperativity and reactivity allow the precise and controlled delivery of therapeutic activity to cancer sites in a stimuli-responsive manner, thus improving the therapeutic outcome while minimizing the treatment-associated side effects. In this review, we have made a comprehensive summary of typical non-covalent interaction modes that may be used for driving the formation of supramolecular nanomedicines and thoroughly discussed their potential roles in directing their engineered stimuli-responsive drug delivery features. However, despite the tremendous clinical success of typical self-assembled nanomedicines such as liposomes, lipid nanoparticles, protein-drug co-assemblies, and micelles, the translation of stimuli-responsive supramolecular nanomedicines has thus far been unsatisfactory, evidenced by the recent failure of thermoresponsive DOX-loaded liposomes (ThermoDox). Indeed, the implementation of supramolecular self-assembly principles for developing stimuli-responsive anticancer nanomedicine is still a new field and many translational challenges remain regarding their behaviors and responses to the complex *in vivo* environment.

A major concern over the existing designs of stimuli-responsive supramolecular nanomedicines is whether their multifactorial and elaborated therapeutic activities could overcome the numerous challenges in the complex environment of living organisms. Previous studies collectively reveal that the anticancer performance of nanomedicines is profoundly affected by many factors including routes of administration, dosage, systemic/local pharmacokinetics, and nano-biointeraction with different tissues/cell populations. Moreover, the inclusion of stimuli-responsive features puts up additional challenges regarding their efficacy and safety as it remains questionable if they could maintain their discrimination capacity against alterations in endogenous or exogenous conditions including pH, ROS, enzymes, temperature, etc, when they are tested on more advanced animal models or real-life patients. Current strategies to address these challenges include the introduction of tumor-targeting ligands through covalent chemistries and the development of multi-responsive mechanisms to reduce the risk of non-specific drug delivery, although their efficacy remains to be investigated under clinically relevant conditions.

It is noteworthy that the interaction modes between individual components in self-assembled nanosystems are usually not monotonic but multivalent, where multiple non-covalent interaction forces may act in a spontaneous manner to form, maintain, and eventually activate the supramolecular structures. Indeed, the agonism between individual non-covalent interactions may enhance the stability of the supramolecular nanosystems to prevent non-specific activation, while their antagonism could induce their dissociation and therefore trigger the designated therapeutic activities, further contributing to the dynamic and responsive control of these self-assembled nanotherapeutics. However, to realize such synergies would require rational engineering of the molecular components according to the physical/chemical profiles of *in vivo* environment.

As described above, transitional metal ions could form electrostatic complexes with oppositely charged components that lead to the neutralization of their charges. However, even those neutralized ions could still complex with other species through metal-ligand coordination. The coordination bonds could also act as a major impetus to enable the formation of supramolecular materials with well-defined geometries such as polygons, polyhedrons, and prisms or amorphous structures. The coordination center could be either metal ions or inorganic nanoclusters, while the organic linker must contain one or more donor sites with electron-donating atoms or functional groups. Interestingly, the metal-ligand self-assembly strategy offers a practical approach to maintain and optimize the biochemical activity of the metal central species while reducing their potential cytotoxic impact. For instance, Li et al. reported that Fe^2+^ ions and DNA molecules could form nanospheres with precise size and composition through coordination-driven self-assembly, which presented a significant advantage for tumor-targeted drug delivery.^137^ Ren et al. exploited the strong metal-polyphenol coordination interaction between phenolic platinum (IV) prodrugs and polyphenol-modified block copolymers to obtain bioresponsive nanomedicines, which could not only reduce the systemic toxicity of cisplatin but also improve the chemotherapeutic and chemodynamic efficacy against tumor cells.^138^ Current evidence demonstrates that metal-ligand coordination self-assemblies have great clinical significance for both therapeutic and diagnostic applications, although the pharmacokinetic properties of these organometallic agents still need to be comprehensively investigated in human systems.

Another interesting topic in the development of supramolecular antitumor nanomedicine is the carrier-free self-assembled nanotherapeutics, which excludes the usage of additional excipients while improving the overall pharmacokinetic profiles of the therapeutic agents. The carrier-free drug nanoassemblies could be facilely prepared through the non-covalent interaction-driven assembly of self-complexable drug molecules, drug mixtures, or bioresponsive drug-drug conjugates. The primary benefits of carrier-free drug nanoassemblies include maximized drug loading capacity, easier formulation process, improved blood circulation stability, tailorable tumor responsiveness, and lower safety risks due to avoidance of excipients. For instance, Liang et al. conjugated hydrophobic camptothecin and hydrophilic fluoropyrimidine derivative onto multivalent pentaerythritol via hydrolyzable ester linkers, and the resultant molecules could self-assemble into liposome-like nanocapsules for efficient tumor-targeted drug delivery.^139^ Wang et al. reported that employing disulfide bond to connect hydrophobic DOX molecules (DOX-S-S-DOX) could balance the competition between intermolecular forces and the resultant DOX-S-S-DOX molecules could self-assemble into nanoparticles in aqueous environment.^140^ Importantly, major issues associated with the development of carrier-free drug nanoassemblies are the selection of proper drug combinations, optimal dose ratio of individual therapeutic agents, blood circulation stability, uptake mechanism by tumor cells and the eventual activation of their therapeutic activities, warranting further evaluations in these aspects on clinically relevant models.

The rich source of self-assemblies in living organisms offers a promising alternative to develop novel supramolecular drug delivery systems with intrinsic tumor specificity and stimuli-responsiveness. Typical examples in this context include exosomes, cell membranes, and peptides. For instance, Nie et al. developed nanoengineered exosomes by conjugating anti-CD47 antibody (aCD47) and anti-signal regulatory protein alpha (SIRPα) antibody linked with pH-sensitive benzoic-imine bonds onto M1 macrophage exosomes via click reaction,^141^ which could impair the immunosuppression mechanisms in TME to enhance the efficacy of cytotoxic T cells. Nie et al. employed cancer cell membrane to encapsulate MSN-supported PEGylated liposomes, which could undergo cell-like spherical-to-ellipsoidal transformation to penetrate deep into tumor tissues, followed by selective fusion with tumor cell membrane through homotypic targeting.^142^ Zhou et al. synthesized two d-tetrapeptide with phosphotyrosine residues and naphthyl-capped N-terminals, which could be dephosphorylated by tumor cell-secreted alkaline phosphatases and self-assemble into nanofibers, leading to efficient tumor cell apoptosis after the subsequent uptake.^143^

### Limitations of the study

The majority of the current studies in the area of stimuli-responsive supramolecular nanomaterials are still in the proof-of-concept stage and much of their mechanical, pharmacokinetic, and safety details remain to be elucidated, presenting a major limitation to their future development. Although most of the reported supramolecular drug delivery systems are fabricated using biocompatible precursors, this does not guarantee their complete safety *in vivo* as the chemical modification and drug incorporation may significantly alter their biochemical profiles and evoke undesirable cytotoxicity or immunogenic reactions. Hence it is important to comprehensively evaluate the impact of these emerging supramolecular nanomaterials on pertinent preclinical and clinical models, as the resulting insights may significantly improve their performance in the complex biological environment of real-life patients.
